# ‘What are you in for?’ The neglected role of the offence in prison sociology

**DOI:** 10.1177/26326663261442087

**Published:** 2026-04-10

**Authors:** Ben Jarman, Alice Ievins, Ben Crewe

**Affiliations:** 1 7423 University of Southampton, UK; 2 4591 University of Liverpool, UK; 3 2152 University of Cambridge, UK

**Keywords:** Adaptation, criminal offences, legitimacy, moral communication, prison sociology, prison ethnography, punishment

## Abstract

Why have prison scholars so seldom discussed what people in prison think about their crimes? This article addresses a striking gap: the omission of the originating reason for incarceration – the offence – from sociological analyses of carceral life. We examine its past treatment in prison scholarship and argue for recentring it. Drawing on three case studies, we show how the offence actively shapes daily experience, institutional engagement, and social dynamics. Our framework explores how attention to the offence can illuminate what prisons *are like*, what they *do* through moral communication, and what they *are for* as sites of punishment. Our analysis suggests that attending to the offence can enrich our understanding of adaptation, compliance, and legitimacy, and more generally add to our understanding of imprisonment.

## Introduction

Prison scholarship has often implied that *why* people are incarcerated – what they have done or are deemed to have done – is irrelevant to the internal dynamics of prisons as institutions. Classic sociological texts treated prisons as analogous to other organisations, overlooking their punitive role (by which we mean their use as an intentionally denunciatory and pain-inflicting response to legally determined criminal wrongdoing). Most obviously, *Asylums* (Goffman, 1961) bundled them with monasteries, boarding schools and military barracks, as ‘total institutions’. *The Society of Captives* likewise saw prisons as systems for ‘total or almost total social control’ (Sykes, 1958: xiv), drawing parallels with concentration camps and gulags which were apt at the time, but which bracket off the prison's task *as a site of punishment* from the subsequent analysis. Foucault's *Discipline and Punish* places punishment centre-stage, but is not really about prisons, so much as what their techniques tell us about a more extensive reorganisation of power around panoptic surveillance and the cultivation of selfhood. ‘Is it surprising’, Foucault (1995: 9) asks, ‘that prisons resemble factories, schools, barracks, hospitals, which all resemble prisons?’ In these studies, the prison is presented generically, not as an institution whose purpose and culture relates distinctively to the reasons people are confined there.

The effect of relegating punishment beneath concerns about power, control and discipline is to neglect one of the prison's most fundamental characteristics. Because people are sent to them as punishment, and often seek to make sense of, or amends for, the acts or convictions that put them there (Herbert, 2019; Irwin, 2009; Schinkel, 2014), prisons are permeated by moral concerns. Indeed, in many jurisdictions, they were designed to encourage moral reflection, an objective unifying their aims, architecture and regimes (O’Donnell, 2016). In other words, offences were once central to how prisons were designed and how they functioned. In recent decades, however, discourses of penal administration have shifted, obscuring the offence's relevance. In many Anglophone jurisdictions, at least, running prisons has become a generic management practice, rather than one with moral dimensions (Feeley and Simon, 1992). Prison management has been framed as a technical and generic task, rather than one with particular normative implications, as if imprisonment has ‘nothing to do with the intended delivery of pain’ (Sparks, 1994: 24).

Sparks’ (1994) proposal to ‘re-moralise’ our understanding of imprisonment has been fruitful. A significant branch of scholarship now addresses normative matters regarding penal legitimacy, focussing especially on prison order and ‘moral performance’ (Liebling and Arnold, 2005; Sparks et al., 1996) and exploring the forms of power that prisons impose, the values by which they operate, and the ways prisoners are treated as moral agents. Such work contributes to broader debates about whether, given the highly unequal distribution of power within prisons, imprisonment is morally defensible. Fewer works address the other dimension which distinguishes imprisonment from ‘ordinary administrative activity’ (Sparks, 1994: 24) – that it is imposed upon people in response to legal and moral breaches that hold meaning for them, and which can animate how the prison operates on them.

We aim to develop this branch of thinking. We agree with Sparks that justifications for punishment must be compelling, and should take account of what imprisonment is like empirically – that is, what prisons do and how they are experienced. But we expand the frame to place the offence alongside power and order, since all three are intertwined in the act, imposition and experience of imprisonment. This makes empirical accounts more descriptively faithful and strengthens theorisation, enabling fuller participation in debates about imprisonment's purposes.

We begin by examining how the offence has featured in prison scholarship – initially as a blunt or latent classificatory variable, but increasingly as something of institutional, social or subjective significance. We consider why researchers have tended not to ask prisoners about the meaning of their offence, nor to centre it analytically. Using three case studies, we demonstrate how attending to the offence illuminates what prisons *are like*, what they *do*, and what they *are for*. Our aim is to explore morally infused issues that are highly salient to how prisoners experience imprisonment, their orientation to the authorities, their reception of forms of moral communication, and broader questions about penal legitimacy.

## Literature review

Laursen and Mjåland (2025) recently described the offence as an ‘absent-presence’ in English and Norwegian prisons: absent, in that their participants felt perplexed by the absence of meaningful communication about it, and yet present, in that a conviction was their single shared characteristic. This characterisation largely holds true for the sociology of imprisonment. We suggest that the offence's role in theorising prisons requires reassessment.

Influential early texts acknowledged offences, but did so mainly to construct analytical groupings – Schrag (1954) found that first-time and non-violent offenders more readily resisted ‘prisonisation’ (Clemmer, 1958), while Irwin and Cressey (1962: 141) distinguished between how professional ‘thieves’ and state-raised ‘convicts’ adapted to the sentence. Both examples theorised adaptative styles via categories derived from conviction types and criminal orientations; neither explored how individuals thus categorised reflected upon the meanings of their convictions. A similar approach persisted later, where scholars concerned with prison order continued to feature the offence as a classificatory variable. For example, Jacobs (1977: 159) noted that an offence-based status system at Stateville had been replaced by a ‘balkanized’ one based on racial and gang affiliations, while Flanagan (1982: 83) suggested murderers were often ‘essentially noncriminal’, and thus distinct from the broader prisoner population.

Some exceptions to this pattern exist, most prominently in relation to the moral distinctions made about prisoners convicted of sexual offences, including their low status within prisoner hierarchies (Cohen and Taylor, 1972), their subjection to intensified staff control (Sparks et al., 1996) and their increased isolation, guilt and shame (Sapsford, 1979). Mathiesen (1965) suggested that such prisoners defended their moral status by engaging in ‘censorious’ critique of prison staff. Meanwhile, *Psychological Survival* (Cohen and Taylor, 1972) addressed guilt and shame only in relation to sex offenders (who it classified as ‘sinners’). Yet a re-analysis of archived materials uncovered expressions of guilt and shame among *all* the Durham prisoners, who felt safe to surface these emotions in Cohen and Taylor's classroom ‘without worrying about being evaluated’ by prison authorities (Fielding and Fielding, 2008: 90). Moral reflection was thus occurring, unprompted by institutional attempts to induce it, and unnoticed by sociologists more interested by authority relations.

Latterly, though, researchers have increasingly recognised the offence's influence over how prisoners experience punishment, understand themselves, and engage in ethical self-formation. This shift partly mirrors the changing character of penal power. In England and Wales, and many other jurisdictions, prisons have become ‘tighter’ (Crewe, 2011; Crewe and Ievins, 2021). Prisoners’ narratives – particularly those relating to their offending – have become objects of discipline. Offending behaviour programmes and risk assessments structure progression and release decisions, and construct prisoners by reference to their offences (Fox, 1999; Lacombe, 2008). Meanwhile, sentence inflation has intensified retributive severity for some categories of offending (Pina-Sánchez et al., 2025).

Perhaps not surprisingly, researchers have become more interested in responsibility and ethical status – that is, in the transmission and reception of censure. Irwin (2009: 43) documented an ‘awakening’ process whereby lifers came to ‘fully appreciate’ that something had been ‘fundamentally wrong with their former behaviour’ (Herbert, 2019; cf. Schinkel, 2014). Meanwhile, narrative criminologists have shown that prisoners shape their offence stories to ‘recreate and reposition themselves as particular kinds of subjects’ (Ugelvik, 2012: 261–262), and to assert masculine legitimacy in the face of discrediting convictions by denigrating peers convicted of sexual offences (Ugelvik, 2015). Ievins and Crewe (2015) also suggest that the targets of such exclusion overlook the convictions of others to reduce the salience of their own.

Recent research has noted that factors including gender (Crewe et al., 2017), age (Tynan, 2019), and offence type (Jarman, 2020) shape the ethical implications of a murder conviction. Murders committed in different circumstances carry varying implications for a subject's felt culpability, while sentences imposed at different ages impact life trajectories and personal narratives differently (Jarman, 2024). These examples focus on lengthy sentences and serious offences, but repeated short sentences also prompt reflections on lives blighted by punishment (Armstrong and Weaver, 2013; Schinkel and Lives Sentenced participants, 2021).

Elsewhere, drawing on normative penal theory (especially Duff, 2001), prisons have been conceptualised as sites of moral communication. Ievins (2023) demonstrates how prisons ‘stain’ their inhabitants, censuring formally via interventions and risk assessments, and informally via spatial segregation, distinctive treatment regimes, and everyday interactions which mark certain people as morally distinct. The resulting censure is often oblique and inconsistent, with prisoners’ interpretations of it diverging from institutional intentions. Similarly, Schinkel (2014) argues that prisoners often do not receive the censure intended by sentencers: the medium of imprisonment distorts the censuring message.

Recent research has also characterised participants’ attitudes to their (often serious) offending. Crewe et al. (2020: 137) found that most life-sentenced prisoners found their offence ‘unambiguously shameful’, affecting their institutional adaptations and social relations throughout their sentences. Research with people imprisoned for homicide in Argentina (Di Marco, 2022) and England (Jarman, 2020) has questioned whether moral reflection inevitably produces remorse, or expressions of responsibility have any stable meaning. Elsewhere (e.g. Crewe and Ievins, 2021; Dagan, 2025; Ievins and Mjåland, 2021), risk management practices are treated as a vector for official communication about the offence. ‘Lateral’ forms of communication among prisoners and informal messages by staff, have also been analysed (Ievins, 2020; Kotova and Akerman, 2022), reminding us that censure can be informal and unofficial. It appears that meaningful reflection on the offence seldom occurs quickly (Crewe et al., 2020; Irwin, 2009), and is often catalysed when a life after prison becomes imaginable, at which point engagement with institutional demands (e.g. for ‘insight’ into the offence) is more strongly incentivised (O’Donnell, 2014; though see also Seeds, 2022 on ‘deeper’ forms of hope).

Adapting Laursen and Mjåland's (2025) term, then, in much 20th-century prison research the offence was *latent*-present: used to signify types of people, but seldom analysed either as a moral category, or as an act holding ethical significance for those categorised by it. Recently, though, attention has shifted to the *subjective meanings* of legal labels – how it feels to be held responsible and how this shapes prison experience. The shift invites us to consider prisons not simply as social settings producing psychological adaptations, but sociologically: as places where the personal and cultural meanings of wrongdoing are negotiated.

An empirical focus on the moral meaning and effects of imprisonment also brings into view normative discussions about the justification and justice of punishment. In most countries, imprisonment is the archetypal form of punishment, and prisons treat reflection on and ‘insight’ into the offence as a desirable response (e.g. Dyke et al., 2020; Paratore, 2016; Shammas, 2019). Thus, a shift towards questions of purpose is an important step, since the *kinds* of insight which might be expected will vary. Communicative theories of retributive punishment (Duff, 2001) can help here, since they insist that punishment expresses communal values and should respect offenders’ moral agency. Although moral censure may be distorted by imprisonment, or go unnoticed (Schinkel, 2014), our contention is that focusing on imprisonment's morally communicative aspects adds a great deal to our understanding of penal legitimacy. This makes it all the more striking that the offence features so little in conventional prison scholarship.

## Researching the offence

Within prisons, someone lacking a story about why they are there, or unduly defensive when asked, is considered by other prisoners to be rather suspect (Crewe, 2009). Yet convention among prison researchers^1^ holds it inappropriate to ask an incarcerated person directly for details of their offence.^2^ This norm is framed primarily as an ethical imperative. Asking people ‘what they are in for’ – it is suggested – risks pathologising or re-traumatising them, compounding their stigmatisation, or conflating the person with their conviction. Alternatively, it taints the interviewer's ability to remain non-judgemental (Stevens, 2013). Thus, while populist discourses censure prisoners as *bad people*, prison researchers have tended to swerve or reject such judgements. This might reflect ideological or political commitment, as where critical scholars direct judgement towards the carceral institution rather than incarcerated people. More often, however, the norm seems likely to have been transmitted via methodological training and supervision, embedding itself in disciplinary practice regardless of individual orientations.

A tendency not to inquire about the offence also reflects the longstanding disciplinary parameters of prison sociology. While normative issues play important if varied roles in penal theory, prison sociology has tended to see them as irrelevant (though see Ievins, 2023; Jarman, 2024; Schinkel, 2014). To some degree, this is defensible. As we note above, offence *type* has been associated with prisoner hierarchies and orientations to authority, but offence *reflections* have seemed somewhat immaterial to core topics of investigation. When researchers study prisoner social relations or penal order, it can appear digressive to discuss prisoners’ thoughts on whether the penalty fits their crime, or their internal battles to maintain a respectable self-image.

Relatedly, interest in prisoners’ interiority has generally been subordinate to interest in their social practices. Canonical texts (e.g. Jacobs, 1977; Mathiesen, 1965) theorised social structures and institutional processes, rather than prisoners’ reflexive existence. Methodologically, the semi-ethnographic emphasis on ‘where the “action” is’ (McDermott and King, 1988) – wings, yards, workshops, and other collective areas – may have missed quieter occurrences in more private spaces. Cells, where prisoners spend much of their time, afford headspace for reflection. The offence may be more present there, but methodologically the cell is less accessible. Contemplation and rumination there leave fewer obvious traces in social life.

Nonetheless, our conjecture is that many prison scholars hear reflections on the implications of prisoners’ offences and convictions but exclude these from their accounts. We find it hard to imagine that anyone interviewing people convicted of serious offences has not been presented with narratives featuring moral emotions like regret, indignation, or defiance; or that those with less serious convictions have not sometimes narrated how and why they landed in their current circumstances, for example, by burgling houses or dealing drugs. If violence and other crimes are communicative, then not enquiring about them means ignoring what they attempted to express. To deflect discussion of the offence might be as disrespectful as to probe too deeply.

How should the well-documented tendency to minimise and neutralise (Sykes and Matza, 1957) affect our interpretation of these accounts? Simply to discredit them as self-serving lies is to misunderstand them. Evaluating narratives purely by their fidelity to the ‘established facts of the case’ unduly privileges the conviction as a complete and authoritative record, while overlooking its significance for the narrator's current situation. Both desistance scholarship (Maruna and Copes, 2005) and narrative criminology (Ugelvik, 2012) recognise that distinguishing between narratives and ‘the truth’ is neither straightforward nor necessarily conducive to understanding. Instead, they should be examined for what they reveal about prisons and punishment. Offence narratives can be understood as moral sense-making: preserving self-worth, making sense of the past, and navigating censure.

Our case studies come from projects which involved lengthy interviews, some over multiple sessions or following a sustained presence in the prison (see Crewe et al., 2020, 2023; Jarman, 2024 for methodological details). While they focused directly on the offence to differing degrees, all three projects employed semi-structured interviews that allowed participants to narrate their experiences of imprisonment and relationship to their convictions. All three asked some questions about the offence and/or conviction and how these impacted people's sense of self, the prison's social world, and the extent to which they were a target of power.

We all felt that participants’ willingness to discuss these matters required us to avoid displaying the shock or revulsion some seemed to anticipate. Existing publications on the ethical considerations involved in interviewing people convicted of serious (and particularly sexual) crimes often focuses on the opposite moral threat: collusion with morally unacceptable viewpoints or factually incorrect accounts (Blagden and Pemberton, 2010; Digard, 2010; Waldram, 2007). We have discussed these dilemmas elsewhere (Ievins, 2023: 33–36; Jarman, 2024: 66–77), but they take a specific form when the research investigates not what actually happened, but how people reflect on it and its role in the prison. Researchers should neither entangle themselves in inappropriate side-taking, nor mislead participants by implying greater sympathy than they feel (Crewe and Ievins, 2015; Malcolm, 1990). However, that need not stop us from recognising the continuing effect of the offence in their lives, via the sense they make from it.

Our case studies derive initially from separate analytical work carried out by each author. Having independently observed that the offence featured little in prison sociology despite its significance in our own data, and each discussed this in our own work (Crewe et al., 2020; Ievins, 2023; Jarman, 2020), we collaborated to address the gap more directly. To do so, we selected cases that were emblematic, in that they illuminated the analytical framework we propose here.

Our original studies employed systematic qualitative analysis and followed similar coding procedures (using NVivo in two cases, Atlas.ti in one). Each developed hierarchical coding schemes that combined descriptive codes (e.g. ‘the offence’, ‘family relationships’) and conceptual codes (e.g. ‘guilt and shame’, ‘moral evaluation’), which were applied both deductively and inductively. For this article, we drew on these existing analyses to identify individuals for whom reflection on the offence seemed especially salient, as evidenced through relevant codes (e.g. ‘feeling responsible’, ‘being labelled’). We then reflected on how the offence seemed to have shaped each person's attitudes towards their wider prison experiences.

The first case study derives from ethnographic research in a medium-security English prison for men convicted of sex offences, part of a larger research programme comparing imprisonment in England and Wales and Norway (see Crewe et al., 2023). Extended observation and 45 interviews (averaging 3 h) explored how penal power shapes social relations, with attention to shame, guilt and conviction legitimacy.

The second case study comes from an interview-based study of 146 men and women serving life sentences for murder, convicted aged 25 or under (Crewe et al., 2020). Interviews, averaging two hours, did not seek offence details, but these were often volunteered, and interviewers engaged responsively, exploring orientations towards the offence, conviction and sentence.

The third case study is drawn from a study of 48 life-sentenced men in two English prisons: a closed, high-security long-term prison, and an open prison tasked with resettlement and release (Jarman, 2024). All shared a murder conviction but varied widely in age, sentence length, time served, age at conviction, and offence and pre-prison circumstances. Interviews averaging four hours were supplemented by document analysis.

## Case studies: The offence in three dimensions

As noted above, our case studies are selected for analytical clarity. They show how considering the offence helps us more fully theorise, in three dimensions, what prison is *like*; what prisons attempt to *do*; and what prisons are *for*.

The first dimension – *what prison is like* – examines the phenomenological and social consequences of the offence. We attend both to objective features of the penal setting, and to what prisoners find subjectively important. For example, the ‘moral weight’ (Hulley, 2025) and personal significance of a particular offence might generate distinct patterns of self-understanding, adaptation, and social interaction. All of these are significant topics in orthodox prison sociology, but we believe they are mediated by reflection upon what the offence and/or conviction mean, subjectively. Indeed, we contend that these can be *the* major mental preoccupations for some people.

The second dimension examines *what prisons do*, in communicating with people and attempting to mould their reflections on their convictions. Here, we ask what messages prisons transmit about particular offences, and how these are conveyed. They include both explicit communications, such as those occurring via therapeutic intervention or risk assessment, and implicit communications embedded in everyday practices and interactions. Messages are also carried by material practices of treatment and intervention, from staff conduct to direct engagement with prisoners’ offending behaviour. Theoretically, this raises questions about how prisoners interpret the often oblique and inconsistent messages they receive and navigate the various attempts by institutions to influence their attitudes, thinking, and behaviour.

The third dimension examines *what prisons are for*. This is a question of purpose and aims, and situates moral communication within a wider context. It connects to how we theorise prison legitimacy, and to broader questions about whether imprisonment delivers something more salutary than mere pain, and how its harms can be justified. Such justifications are shaped by the aims of sentencing, and the extent to which these are realised. This is because different penal logics – whether retributive, rehabilitative, deterrent, or incapacitative – imply different messages about wrongdoing. Different penal logics also co-exist within the same institutional context, producing tensions and contradictions. Still, both the content of censure, and the efficacy of prisons in delivering it, are amenable to empirical investigation. This places the offence – how it is understood by both the individual and the institution – centre-stage.

### Mick

Mick,^3^ held in a medium-security prison for men convicted of sex offences, was heavily involved in the informal prisoner economy, and frequently got into bitter, public arguments with prison staff. When interviewed, however, it became clear that this defiant persona stemmed from his complicated relationship to his imprisonment. When Mick was a child, he was persistently abused by an adult family member, and said that this had involved coercing him (Mick) into his own acts of perpetration. Years later, he reported his abuser to the police, but was ultimately prosecuted for acts which he said he had committed under duress as a child. He was found guilty, and sentenced to a sentence of several years in prison.

Mick's anger had its ‘root’ in ‘what happened when I was a kid’, but since then ‘everything else just piled on top of it’. He had flashbacks of ‘what happened […] every night before I go to bed’, and had ‘really bad trouble sleeping’, and would lie awake thinking about ‘just everything that happened, what's going on, the fact that I’m here now and it really shouldn’t be me’. Now, he was ‘still living around paedophiles again, like I did for most of my childhood’. He was attuned to and disgusted by signs of inappropriate sexual behaviour from prisoners and staff, but the depth of his rage came from what his fellow prisoners represented and reminded him of: ‘I’m angry at all these people who haven’t done nothing to me but I just see them as 1100^4^ of [my abuser] and I can’t get that out of my head’.

He maintained emotional distance, engaging in shallow forms of sociability for trading purposes, but had no friends on the wing, and preferred solitude:

I can’t wait for that door to close of an evening to get away from it all. […] I have to socialise to a certain degree during the day, but it isn’t really socialising. It's getting what I need from these people. […] I put this fake persona on of happy, laughy, jokey, “Oh, you alright?”

He lied to other prisoners about his conviction, telling them he had been falsely accused of sexual assault by his girlfriend because he couldn’t bear the idea of telling someone the truth and them turning out to be a ‘raging paedophile’; he also did his best to ‘switch off’ to conversations about offences. Nevertheless, he maintained there was a moral difference between him and his apparent peers. He described himself as infuriated when he heard other prisoners claim that ‘we’re all in the same boat here’: ‘I think, “We’re not in the fucking same boat here. We might have all ended up on the same desert island but fuck me; I rowed here in a completely different boat to you”’.

His relationship with officers was actively fractious – ‘[they] piss me off on a daily basis’ – and he was especially censorious towards those who did not prevent inappropriate sexual behaviour, or engaged in interactions he perceived as flirtatious or unboundaried. He complied when he wanted to, but cared too little about the prison's incentives for them to work on him:

I don’t like playing the victim, but, I feel really wronged for being here, and feel like the right person hasn’t been brought to justice, so therefore, give me a DVD player, it's not going to make me feel any better. It would kill some time but nothing that they can do or give me is going to make me feel, “Oh, it's okay being here, isn’t it?” Are they going to put [abuser] in prison and make him suffer for what he's done? No, they’re not. Well, then I don’t want to comply with your shitty regime.

Nevertheless, he was open, at least in theory, to engaging in therapeutic work. He was unwilling to participate in the Sex Offender Treatment Programme, which involved discussions of the offence itself – ‘there's no way I’m sitting in a room listening to ten fucking paedophiles talking about what they’ve done’ – but he wanted to do ‘anything that will help me deal with this’. In his previous prison, he had seen a counsellor, who had helped him begin to ‘come to terms with the fact that maybe I’m not fully to blame here’. In his current prison, he received no therapeutic or offence-related support, and could not even be properly risk assessed because of the age at which the offence was committed. This meant he had no pathway to demonstrate reduced risk and potentially ameliorate his conditions after release.

Despite his significant frustration with prisoners and prison staff, Mick insisted that what really mattered was the damage that had been done to his family. Prison might be difficult to deal with, but it was bearable. What was harder to cope with was the fact that he was unable to contact the family members he had hurt: ‘I can deal with being in here. I can deal with this shitty routine and the knobhead prison officers and fucking raging paedophiles and stuff that are around but the one thing I can’t deal with is that’. Alongside his real anger at the injustice of his situation were profound feelings of guilt about what he had been involved in. Being in prison, and the conviction that came with it, was a deeply painful because it was unfair and staining, but also because it confirmed what he feared about himself. He had always believed ‘that I am a horrible person and that I am a bad person because I didn’t protect’ people, and the conviction ‘just concretes what I thought of myself […] and makes it true. I think that it's broken me as a person’. Overall, then, while Mick may have appeared non-compliant or a troublemaker, he was preoccupied by the felt injustice and shame of his incarceration, a sentiment which seeped into and infected how he interacted with prison powerholders.

### Chris

Chris was an intimidating presence: muscular, taciturn and with an air of defensive intensity. He described relatively little about his childhood, but said he had been exposed to considerable physical and sexual violence. As a result, he had been an angry young man who had resorted to drugs to cope with and mask his emotions: ‘All my childhood was drugs’. He had been convicted of a murder with a sexual component, and was saturated with feelings of shame: ‘It eats at me every day’. After his arrest, he had pled guilty immediately and made no attempt to minimise his culpability. Sentiments of remorse and self-loathing dominated the interview, just as they governed his experience of imprisonment.

Chris was a ‘loner’, with just a couple of loose associates, whom it had taken him months to first talk to. He did not need other people, he said, and struggled with social situations: ‘in an empty room it’ll feel crowded to me, because I’ve got so much going on upstairs’. He had no involvement in illicit activity, having given up drink and drugs because of their role in his offence: ‘As soon as I committed my crime my head went … gone. I never touched anything again’.

His coping mechanisms were also relatively solitary, shaped by his feelings about his offence. He exercised compulsively, using a rowing machine to ‘take my mind off it’ and ensure that, at the end of each day, he was too exhausted to ruminate on his crime. Day-to-day, he was preoccupied with obtaining sufficient sleep and protein to help him build muscle. Alongside physical activity, Chris liked to paint, which he said was a way of processing and expressing his feelings. He had converted to Christianity during his sentence, and this provided further emotional relief: ‘that's what I was looking for, peace and forgiveness’.

Despite his psychological torment, Chris had not asked for help from staff, stating that he had no faith that they would want to support him: ‘It's a bad crime […] why would you want to help a convict who's committed a serious crime?’ His declaration that he did not trust staff, that it was ‘us and them’, and that he did not need any help betrayed an underlying sense that he was unworthy of support and humanity. He assumed that anyone who read details of his offence in his file would hate him, and therefore pre-empted negative responses by telling staff: ‘You don’t even have to try with me, just leave me be’.

Chris' orientation to his sentence was normative but disengaged acceptance. He experienced both the sentence and his punishment as entirely legitimate. Yet the prison was virtually irrelevant, either as a communicative institution or as a site of intervention. That is, given his profound feelings of shame, external moral censure was redundant: ‘I punish myself more than any of these can punish me’. He harboured no feelings of resentment about his sentence length, commenting on several occasions that he ‘deserved what I got’. This sense that he warranted punishment – and that he was morally worthless, almost a non-person – was expressed in a general fatalism about his treatment (‘they’re here to punish us [and] I don’t mind them punishing us’), the passage of time (‘every day comes, every day goes – it's all part of the punishment’), what was written about him on file, and his progression through the system. ‘I just go with the flow’, he commented: ‘whatever happens, happens. I just see it as it's all part of the sentence’. He declared himself unconcerned by how his time in prison might affect him – ‘Like I say, it is what it is’ – and, due to the nature of his offence, felt he had no right to judge others.

Chris did not believe he could ever move on from what he had done. He was haunted by intrusive recollections of his offence, and, when asked about the hardest time during his sentence, said: ‘Maybe just the nightmares, stuff like that. […] I wouldn’t say anything else has caused a problem’. He had not yet undertaken any courses because he felt he would not be able to cope with having to think about his own crime or with exposure to ‘graphic detail’ about other people's offences: ‘when people are talking about their crimes I’m seeing their crime in my head, and it kills me. […] I can see my own, I don’t want to see theirs, you know’. Emotionally, he was extremely tightly wound.

Despite feeling he could never ‘put it right’, Chris described practices consistent with a desire to demonstrate ethicality. For example, although he generally distanced himself from prison officers, he sometimes informed them about issues and potential incidents on his wing. His justification (for what other prisoners would consider ‘snitching’) was to avert it being on his conscience were someone to get hurt. Likewise, he reported getting involved in disputes not out of loyalty to others, but because he wanted to avert violence: ‘if anyone tries to attack anyone, then that's when I go in, because silliness comes of silliness’. Despite this drive to compensate for his offence through everyday moral action, he described himself as having no goals and said he was living day-to-day, without contemplating the possibility of release: ‘I’m not really interested at the minute’. The offence, and his feelings of deep shame about it, dominated his interior life, orientation to the sentence and social relations, in ways that trumped and made redundant penal censure.

### Nicholas

Nicholas had recently arrived in an open prison, having spent many years in maximum-security custody. Though he had previously mistrusted staff and feared other prisoners, his attitudes now were noticeably positive. For example, he remarked that he had been ‘very, very, very, very lucky’ in the staff who had worked with him, and described sharing jokes with officers. At the system's shallow end, he was struck by the degree to which the ‘bond between [prisoners in] closed conditions’ had been superseded by more individualised thinking, as they turned towards resettlement goals and the post-prison future. Indeed, his own mind was increasingly doing the same:

Am I going to have a good quality of life if I am released next year? I will be [in my forties] […] Am I going to have enough time to get married? Am I going to have enough time to have kids? […] I would never, ever treat my wife or children the way that my dad treated me.

In childhood, Nicholas had suffered physical, sexual, and emotional abuse inside and outside the home. His first attempt to talk about any of this had been in the context of a police interview: he offered a partial disclosure to the detectives who would shortly charge him with murder, but no further action resulted. Two decades on, he verbalised the emotions he had once acted out through violence:

There was so much aggression when I finally committed those offences. But I think [it was] loneliness [that] resulted in that anger […] Not being seen, and then suppressing feelings about that, and then continuing not to be seen, just getting sort of angrier and angrier about it […] I was emotionally withdrawn. Lonely, confused. And still very young […] There wasn’t that feeling of sadness. Or shame. Or fear […] There was no attachment to it or to the consequences of it at all.

Nicholas saw that the gravity of his actions warranted censure. But at the time, his disclosure having resulted in no further action, he reverted outwardly to detachment and denial. As a result, he had spent the first decade or more of the sentence living in two parallel realities. In one, he had learned to cope and adjust. In young offender institutions, terrified of wing life, he had stayed in his cell whenever possible. Later, in adult prisons, things ‘started to move upwards’. Yet this outward adaptation had masked his inability to accept what he had done. There was no *inward* adjustment, still less any reconciliation, to the formal responsibility imposed by his conviction. He could only retreat into fantasy: ‘It was very easy to put myself […] somewhere else […] to blank out what I had done’. Eventually, however, the dissonance between inner and outer life more and more resembled a problem:

If you allow yourself to be switched off 24 hours a day from reality […] eventually it gets to an extent where you find yourself too far gone. It becomes hard to bring yourself back to reality. I don’t think that that changed for me for [more than ten years].

Thus, while a process of adaptation appeared to be ongoing, something else more personal and related to the offence was *also* shaping Nicholas' engagement with the sentence:

I wasn’t in healthcare [anymore]. I was on normal location. I wasn’t as medicated […] I was comfortable on the wing, around people […] [But] I still had that ongoing battle, sort of thing, with that detachment. […] I was just, you know, fighting with myself on a day-to-day basis and just going through knowing that [what was] in my mind was something else compared to what was going on for real.

The challenge he described was to find some way to account to others – not just to himself – for his conviction. Nicholas' disclosures to detectives might be seen as an early, halting attempt. He revisited the disclosures during several years in a prison unit whose therapeutic ethos, he said, meant there was ‘no way of hiding’. It offered a framing of his story which omitted neither his offending nor the soil it had grown in:

It enabled me to see […] how those childhood events […] shaped that person I’d become. […] It's hard to sit there, in a group, and see something being read out on a board, in front of everybody, and actually say, “Yes, that person that's on there that you’re reading out is really horrible. […] That's not a nice person. […] But that person is me.”

Nicholas expressed happiness at his formal progression to the verge of release. Along with his improving family relationships, he counted this as a real positive. But the deeper gain, he made clear, was a more ‘normal’ relationship with himself:

*Interviewer: *Have you gained anything that matters to you since you’ve been in prison?

*Nicholas: *Yes. My religion. [Long pause] My family. Myself.

*Interviewer: *You’ve gained yourself?

*Nicholas: *Yes. Going along those lines that … I matter as well. […] That is what I’m referring to. […] After I went to the [therapeutic] unit […] it was all about me, and about accepting me for who I was.

Although he was pointing here to subjective benefits, Nicholas' orientation was far from solipsistic, and in fact evinced a form of accountability. He believed victims should be able to demand and receive explanations, and regretted that the parole process had not afforded opportunities for such dialogue:

I also look at it from other people's point of view. […] My victim's family […] has that involvement with the victim liaison officer. They went to my hearing when the parole [board] sent me to open conditions. So, although they don’t have contact with me, and they never probably will have contact with me because they don’t wish to know me […] they still don’t know why I’ve done what I done.

For Nicholas, then, the offence was not a static fact to be managed or concealed, but a living problem requiring ongoing moral work; it shaped, but also exceeded, questions of adjustment or compliance.

## Discussion

Regarding our first analytical dimension, attending to the offence clarifies *what imprisonment is like*, enriching accounts of adaptation, compliance, staff-prisoner relationships, and social relations. Conventional accounts (e.g. Crewe, 2009; Skarbek, 2020; Sykes, 1958) typically depict prisoner social dynamics as being driven by urgent needs – for example, to mitigate deprivation, secure safety, defend interests, or protect identity. In this paradigm, the solutions offered by social interaction impel prisoners’ actions in almost mechanical ways. This framework is persuasive but reductive, overlooking how internal moral and psychic drives can also generate action.

To develop this point, the primary preoccupation for many prisoners may be psychological rather than social survival: an *internal* struggle relating to the moral implications of serious offending. Grasping *what imprisonment is like* for Mick, Chris, or Nicholas – what dominates mental headspace – requires centring the offence. For all three, the importance of the prisoner social world is subordinate to that of coming to terms with feelings of shame and bewilderment relating to the offence. This was most strongly exemplified by Nicholas, who stated explicitly that his outward adjustment had concealed inward turbulence.

This inner–outer contrast can enrich our understanding of topics of longstanding interest. For example, staff–prisoner relationships have typically been understood through criminal codes, authority orientations, and power disparities (e.g. Cohen and Taylor, 1972; Drake, 2012; Irwin and Cressey, 1962). Yet Mick and Chris show that prisoners who distance themselves from officers (or other staff) might have in mind how those staff members represent certain feelings about their convictions, or about how the criminal justice system has interpreted their acts. Prison staff do not simply represent state repression (e.g. Irwin, 1980; Jacobs, 1977), nor act simply as agents of discipline and control (Carroll, 1974; Drake, 2012). They also symbolise *punishment*: if prisoners perceive staff to be the deliverers of a deserved (or harsh) penalty, their orientations towards them may be altered as a result.

Compliance, including engagement with programmes, is shaped by offence-related sentiments – hence Mick's tense relationship with staff and refusal to conform to an undeserved regime, Chris' general disengagement and resistance to activities he expected to vivify his intrusive thoughts, and Nicholas' difficulties accounting for his past actions without therapeutic tutelage. Just as prisoners’ (non)compliance can be motivated by the perceived legitimacy of their treatment (Liebling and Arnold, 2005; Sparks et al., 1996), so too it can be shaped by feelings about the offence for which they are imprisoned.

The topic of social relations illustrates the same point. How our examples chose to interact with their peers, involve themselves in prisoner society, or self-isolate, cannot be attributed only to their ‘problem-solving’. Their choices also reflect comparative moral evaluations prompted by the offence and the conviction, filtered through emotions like disgust (Mick), the capacity to handle ruminative turmoil (Chris), or the challenges of accounting to others for one's actions (Nicholas). Meanwhile, conventional ‘coping mechanisms’, such as Chris' commitment to faith, exercise and art, can be reinterpreted as ‘ethical projects’ – a means to grapple with emotions summoned by the offence, and not mere ‘escape activities’ (Goffman, 1961) or outlets for frustration (Laws, 2022).

Regarding our second analytical dimension, considering the offence clarifies *what prisons do*. Some of their operations are directly and obviously organised around the offence, particularly when they attempt to mould how people reflect on it. Such reflections might be an explicit target of power – as when prisons seek to equip people with ‘skills’ which will help them manage their risk in future – and are sometimes the basis of organisational categorisation. For example, Mick was categorised as and held among ‘sex offenders’, but felt morally distinct from this classification. In other words, prisons are morally communicative institutions – a role and function which has not been properly explained in previous accounts (though see Ievins, 2023; Schinkel, 2014).

Even when the offence is not explicitly threaded into the operations of power, the fact of being in prison, and of how prisons are organised, influences how people reflect on and relate to their conviction. Our case studies show how moral and penal censure interacts with the offence, a process with significant personal consequences. Nicholas offers a rare example of how imprisonment can facilitate a more productive relationship with the self, in his case arising from being supported to ‘sit with’ his culpability.^5^ Mick, by contrast, felt imprisonment might potentially have helped him ‘come to terms with everything that's happened’, but for experiences of misrecognition and unmerited punishment which blocked this outcome. He oscillated between bitter resentment and profound shame prompted by the harms for which he did feel responsible. Chris, meanwhile, assumed that the system judged him harshly, in line with his own self-evaluation. Such was the depth of his self-disgust that he neither needed nor wanted external engagement, and could perceive no means by which to digest his feelings of shame.

Our case studies also illuminate fundamental tensions in how contemporary punishment actually operates, versus how it justifies itself. This unavoidably brings into view the third dimension of our framework: questions of *what prisons are for* and whether they fulfil their objectives. Seeing prisons as morally communicative institutions involves seeing that they contribute to a wider punitive ritual (Durkheim, 1973), which signals disapproval and affirms values essential to collective life. It is a paradox of the modern prison that it has obscured punishment – a hitherto public and participatory procedure – through enclosure, bureaucratisation and legalisation (Garland, 1990). Attending to the moral messages prisons convey, however, casts light on their punitive functions. Viewed thus, imprisonment is not an ‘ordinary administrative activity’ (Sparks, 1994: 24) but rather one subject to more stringent justification and different forms of legitimacy.

Our case studies offer evidence about how individual accountability – broadly understood to mean ‘taking full responsibility for oneself and one's harmful actions’ – is pursued in practice. Advocates of different penal logics place differing weight on this goal. Some modern retributivists believe that holding people accountable for their wrongful acts, and giving them the chance to take full responsibility, is a justifiable goal of punishment (Duff, 2001; Tasioulas, 2007); critics, on the other hand, view the pursuit of accountability as a dangerous, futile, and backward-looking overuse of state power (Von Hirsch, 2003).

Chris' and Mick's stories illustrate some dangers of the exclusive pursuit of accountability – a goal which, with Chris, did not require punishment, and with Mick, did not tally with the moral complexity of his conviction. Nicholas, however, illustrates that, under very specific circumstances, retributive approaches might be integrated with rehabilitative ones. Therapeutic intervention had allowed him to see *both* that he was culpable *and* that ‘I matter as well’ – a form of accountability approaching what McNeill (2012: 15) terms ‘moral rehabilitation’. Nicholas' clearly articulated concern for his victims was striking, as was his view that his obligations to them were not fully discharged. Like Mick, he was troubled that imprisonment denied him the opportunity to apologise to and make amends with the people he had harmed. Both men suffered because their punishment felt like an incomplete ritual, bringing them to the point where they desired repair, but were unable to actually achieve it. Their examples reveal how contemporary imprisonment often initiates – without completing – the moral work of censure that punishment is claimed to perform. Seen in this way, prisons thwart the punitive ritual which is claimed to justify their use in the first place.

## Conclusion

We have argued in this article that prison sociology's traditional concerns are enriched considerably by considering the offence. It illuminates what preoccupies prisoners, the moral evaluations shaping their actions, and how prisons manage them as people defined by a conviction. It is startling that so little research has closely studied these matters, documenting how the subjective moral implications of criminal offences infuse the carceral lifeworld. The prison's role *as a punitive institution* should be more central to how we theorise it. Prisons deliberately impose suffering on people whose imputed wrongdoing is deemed to justify it. In wrestling with what they deserve, those people engage with what it means to be punished in a range of ways, including by punishing themselves.

The interaction between institutional and individual reckonings produces much of the prison's ‘moral intensity’ (Liebling, 2025: 1), yet its moral effects are often ‘assumed rather than empirically studied’ (2025: 2). Some such effects – those produced by the loss of liberty and its collateral consequences – are well documented. But the existential texture produced by ‘darkness, power and deprivation’ (2025: 1) is overlain by an additional layer of moral sentiment relating to ‘the offence’. Bringing this layer into focus means participating in normative discussions: not to make declarations, necessarily, but to bring empirical complexity to an impoverished public discourse that swings between vindictive moralising (‘bad places for bad people’) and naïve fantasies of rehabilitation (‘using prisons to reduce reoffending’). Such representations contribute to a seriously unhelpful politics of punishment, because they ‘roam free in a realm of imagination and mythmaking with only the most tenuous connection to what happens inside the system’ (Sparks, 1994: 24). Neither vindictive moralising nor rehabilitation fantasies capture the complexity of our case studies, which show ethical work that is often self-initiated, incomplete, and shaped by offence-specific meanings.

Our cases concern offences with an intense ‘moral weight’ (Hulley, 2025), in that the men we discuss here had all been imprisoned for serious violent or sexual offences. While this might appear to limit the applicability of our argument, our analytical framework can extend more broadly.^6^ Across our studies, the offence was always of some relevance to sentenced prisoners, since regardless of how individuals relate to it psychologically, the conviction always defines the severity of the sentence and shapes progression pathways. Its *subjective* significance, though, varied considerably. We acknowledge that someone serving a 10th short sentence for burglary might not ruminate on one specific offence to an extent that subjugates their other concerns. Hence a less ‘weighty’ conviction might not always (in our framework) shape *what prison is like*. Nonetheless, it will always be analytically significant for understanding how prisons communicate censure (*what they do*), and what purposes they serve (*what they are for*). Moreover, less serious convictions may still hold subjective significance: people serving repeated short sentences grapple with and act upon feelings of shame, resentment and existential futility (e.g. Crewe, 2009; Schinkel and Lives Sentenced participants, 2021); and when accumulated penal harms vastly exceed the gravity of any individual conviction, the very meaninglessness people experience might also reveal something important about *what prisons are for*.

In our view, the extent to which different (and not only serious) offences shape prison experiences across our three dimensions – phenomenologically, institutionally, and purposively – is an empirical question worthy of systematic investigation. We urge other scholars to theorise the prison with these issues in mind, and offer a framework to guide future research. It moves beyond the tendency to treat offences as mere classificatory variables, recognising instead that their subjective meanings shape the prison experience powerfully. Scholarship already moving in this direction requires fuller development.

## Notes

1. The norm is often not explicit in methodological reflections, but we have discussed it with many other prison researchers and are confident that how we have articulated it here is consistent with the messages that most junior scholars absorb.

2. There is, of course, research which interviews people in prison *specifically about their offending* and the nature of crime and criminality, but we count this as research done within prisons rather than research about imprisonment.

3. All names are pseudonyms, and we have changed any potentially identifying features.

4. 1100: the population, approximately, of the prison where Mick was held.

5. An important precondition for this change was its setting: a prison unit characterised by supportive, and yet demanding, forms of intervention.

6. We thank an anonymous reviewer for pushing us to strengthen this point.
